# Reciprocal brain–behavior associations in gambling disorder revealed by theta cordance and directional predictive modeling

**DOI:** 10.3389/fnbeh.2026.1707261

**Published:** 2026-09-03

**Authors:** Eda Yılmazer, Metin Çınaroğlu, Selami Varol Ülker, Sultan Tarlacı

**Affiliations:** 1 Psychology Department, Faculty of Arts and Science, Haliç University, İstanbul, Türkiye; 2 Department of Psychology, Faculty of Administrative, Business and Social Sciences, Istanbul Nisantasi University, İstanbul, Türkiye; 3 Psychology Department, Faculty of Humanities and Social Science, Üsküdar University, İstanbul, Türkiye; 4 Medical School, Üsküdar University, İstanbul, Türkiye

**Keywords:** behavioral addiction, bidirectional brain–behavior interactions, gambling disorder, Granger causality, theta cordance

## Abstract

**Background:**

Gambling disorder (GD) is a behavioral addiction characterized by impaired impulse control and fronto-limbic cortical dysfunction. EEG theta cordance, which integrates absolute and relative power to index regional cerebral perfusion and metabolism, has not previously been combined with directional predictive modeling in behavioral addiction research.

**Objective:**

To compare regional resting-state theta cordance between GD patients and healthy controls (HCs), and to examine directional predictive associations between cortical cordance and clinical severity indices.

**Methods:**

In this retrospective observational study, 28 male patients with DSM-5 GD and 29 male HCs underwent 21-channel resting-state EEG (eyes-closed, 3-min artifact-free epochs; 4–8 Hz). Theta cordance was computed using the Leuchter algorithm and averaged into eight regions of interest. Clinical measures included the South Oaks Gambling Screen (SOGS), Beck Depression Inventory-II, Beck Anxiety Inventory, and illness duration. Group differences were tested with independent-samples *t*-tests (with Bayesian complements) and Pearson correlations; an exploratory vector autoregressive (VAR)-based directional predictive framework was applied to cross-sectional observations.

**Results:**

GD patients showed significantly lower central (0.03 ± 1.18 vs. 1.25 ± 1.23; *t*(55) = −3.79, *p* < 0.001, *d* = −1.00) and higher left parietal cordance (*t*(55) = 2.12, *p* = 0.039, *d* = 0.56), with trend-level occipital (*p* = 0.058) and left temporal (*p* = 0.067) differences. No cordance–clinical correlations were significant (all *p* > 0.10). VAR-based analysis identified asymmetric predictive associations: prefrontal (*F* = 4.60, *p* = 0.042) and frontocentral cordance (*F* = 6.65, *p* = 0.017) with SOGS; central cordance with illness duration (*F* = 5.77, *p* = 0.024) and right temporal cordance (*F* = 5.53, *p* = 0.027); and reciprocal clinical-to-cortical associations (SOGS→central, *F* = 4.82, *p* = 0.038; duration→right temporal, *F* = 4.53, *p* = 0.044). HCs showed temporo-occipital directional patterns without clinical correlates.

**Conclusion:**

Cortical theta cordance and clinical severity in GD appear reciprocally associated within an exploratory directional framework. Findings are preliminary given the modest, male-only, single-site sample and cross-sectional design, and require longitudinal confirmation.

## Introduction

Gambling disorder is a behavioral addiction characterized by compulsive betting despite harmful consequences (Yau and Potenza, 2015). It shares neurobiological features with substance addictions, including dysregulation of brain regions involved in impulse control and rewards processing (Balodis and Potenza, 2020). Neuroimaging and EEG studies have identified functional alterations especially in frontal and temporal (limbic) regions of the brain (Clark et al., 2019). For instance, pathological gamblers often exhibit dysfunction in the prefrontal cortex (notably orbitofrontal areas) and insula (a cortex buried within the temporal lobe) (Zois et al., 2017). Such abnormalities can manifest as reduced frontal activity during decision-making, which is thought to underlie impaired executive control and heightened impulsivity in gambling disorder (Ioannidis et al., 2019). These observations align with a growing consensus that gambling disorder involves fronto-limbic circuit disturbances akin to those seen in drug addictions (Ishikawa et al., 2025).

One approach to probing these neural disturbances is through quantitative EEG (qEEG) measures (Kim et al., 2018). However, conventional EEG spectral power metrics have notable limitations in mapping brain dysfunction (Darvas et al., 2004). Absolute power (the raw power in a given frequency at an electrode) and relative power (the power in that band relative to total EEG power) each capture different aspects of neural activity, but alone they provide an incomplete picture (Hughes and John, 1999). Absolute power can be confounded by global shifts in signal amplitude, whereas relative power normalizes out global effects but may obscure local changes (Zheng et al., 2018). To overcome these limitations, Leuchter et al. (1994a) developed the EEG cordance method, which integrates both absolute and relative power to generate a more physiologically informative index of regional brain activity. The cordance algorithm involves reattributing EEG power spatially and combining transformed absolute and relative values, yielding cordance measures that range along a continuum. Positive cordance (termed “concordance”) indicates a region’s activity pattern is consistent with normal perfusion/metabolic function, while negative cordance (“discordance”) reflects patterns associated with reduced cortical perfusion and metabolism (Leuchter et al., 1994b). Notably, cordance has shown a stronger correlation with cerebral blood flow and metabolism than conventional power measures (Leuchter et al., 1999). In practical terms, cordance is thought to act as a surrogate marker for regional brain perfusion (Hunter et al., 2006), as validated by studies correlating cordance with PET/SPECT imaging (Cook and Leuchter, 1996; Iannaccone et al., 2023; Kamitaki et al., 2021). This makes cordance a promising tool for mapping functional brain alterations in clinical conditions, potentially offering a more sensitive and specific EEG-based biomarker of cortical dysfunction.

Despite the advantages of theta cordance, most EEG studies in gambling disorder have relied on conventional spectral analyses or static connectivity measures, which do not characterize asymmetric predictive relationships among neurophysiological and clinical variables. In the context of addiction, understanding whether alterations in brain activity are associated with changes in clinical severity in a directional manner may provide insights beyond those obtained from cross-sectional correlations alone. To explore these relationships, the present study employed a vector autoregressive (VAR)-based directional predictive modeling framework inspired by the Granger predictive approach (Cekic et al., 2018; Friston et al., 2013; Seth et al., 2015). Although classical Granger causality is designed for temporally ordered time-series data, the present implementation was applied to cross-sectional observations as an exploratory statistical method to identify asymmetric predictive dependencies among EEG theta cordance measures and clinical indices. Consequently, the resulting directional relationships should not be interpreted as evidence of temporal or causal effects. Rather, this framework was used to examine whether cortical cordance values, as proxies of regional brain function, exhibited directional predictive associations with gambling disorder severity and illness duration beyond conventional contemporaneous correlations. Based on theoretical models of addiction emphasizing reciprocal interactions between neural dysfunction and clinical manifestations, it was hypothesized that frontal cortical abnormalities and gambling severity would demonstrate directional predictive associations consistent with a self-reinforcing brain–behavior framework.

This study represents a novel application of EEG theta cordance combined with an exploratory VAR-based directional predictive modeling approach in behavioral addiction research. By extending conventional correlation analyses, this framework provides an initial examination of directional brain–behavior associations in gambling disorder while acknowledging that confirmation of temporal relationships will require longitudinal studies with repeated neurophysiological assessments.

## Materials and methods

### Study design and setting

This retrospective observational study was conducted at NP Hospital İstanbul, a specialized neuropsychiatric facility affiliated with Üsküdar University. Clinical and neurophysiological data were retrieved from patient records and the hospital’s EEG laboratory database. GD patients were identified based on clinical diagnoses established by board-certified psychiatrists and/or neurologists in accordance with DSM-5 criteria. HCs, who underwent EEG assessments as part of routine clinical or research protocols at the same institution, were also included. Extracted datasets comprised demographics, psychiatric comorbidities, disorder duration, psychological assessments, and quantitative EEG theta cordance measures.

### Participants

The final sample included 28 individuals with GD and 29 HCs. All GD patients had artifact-free EEG recordings and complete demographic and clinical information in their medical records. Exclusion criteria for both groups included a history of neurological disorders (e.g., epilepsy, stroke, major head trauma), current substance use disorders other than nicotine, or severe medical conditions that could interfere with EEG recording. HCs were additionally confirmed, through structured clinical interviews and record reviews, to have no lifetime psychiatric disorders, no neurological illness, and no psychoactive medication use.

### Clinical and demographic measures

Demographic information, including age, gender, education level, and family status (such as number of children), was extracted retrospectively from hospital records. Clinical variables for the Gambling Disorder group also included the presence of psychiatric comorbidities and illness duration, expressed in years since onset of problematic gambling behavior. Psychological assessments were obtained using standardized and validated instruments in Turkish. Anxiety symptoms were measured with the Beck Anxiety Inventory (BAI), which is a 21-item self-report questionnaire assessing the severity of clinical anxiety. The Turkish version of the BAI has been shown to possess strong internal consistency (Cronbach’s α = 0.93) and validity in distinguishing anxious from non-anxious patients. Depressive symptoms were assessed with the Beck Depression Inventory-II (BDI-II), a widely used 21-item self-report measure of affective, cognitive, and somatic dimensions of depression. The Turkish adaptation demonstrated high internal consistency (α = 0.89–0.90), strong test–retest reliability (*r* = 0.94), and established cut-off values for minimal, mild, moderate, and severe depression. Gambling severity was evaluated using the South Oaks Gambling Screen (SOGS), a 19-item screening tool that is widely used in both clinical and research settings. The Turkish version of the SOGS has been validated as a reliable and culturally appropriate measure, with excellent test–retest reliability (*r* = 0.95), strong internal consistency (Cronbach’s α = 0.88), and a cut-off score of eight indicating probable pathological gambling.

### EEG data acquisition and preprocessing

Resting-state EEG recordings were obtained using a 21-channel cap conforming to the international 10–20 system. The total recording duration was approximately 10 min, including alternating eyes-open and eyes-closed conditions, as well as photic stimulation blocks. For spectral analysis, only artifact-free eyes-closed segments were retained. Recordings were sampled at 250 Hz and band-pass filtered (0.159–30 Hz), with an additional 50 Hz notch filter applied to eliminate line noise. Segments contaminated by ocular (e.g., blinks, saccades), muscle, or technical artifacts (e.g., electrode pops, movement noise) were visually inspected and removed. Three-minute clean epochs were then subjected to Fast Fourier Transform (FFT) decomposition to obtain absolute and relative power value theta frequency bands (4–8 Hz). Theta power values were averaged across cortical regions of interest (frontal, central, parietal, temporal, occipital) for use in theta cordance computations.

During acquisition, all electrodes were referenced online to linked mastoids (A1+A2). EEG preprocessing and cordance analyses were performed using NeuroGuide software (version 2.5) together with custom scripts implementing the validated Leuchter cordance algorithm. Prior to spectral decomposition and cordance computation, the data were re-referenced to the average reference.

### Theta cordance calculation

Theta cordance values were derived from the preprocessed EEG epochs using the algorithm originally described by Leuchter et al. (1994a). This method integrates absolute and relative power across electrode sites, applies spatial normalization and z-transformation, and combines standardized values to yield a physiologically meaningful index of cortical activity. Positive indices indicate concordance (cordance), whereas negative values indicate discordance. In practice, cordance was calculated using a validated macro implementation of this algorithm in three consecutive steps. First, absolute power values were reattributed to each electrode by averaging power from all bipolar electrode pairs that included that electrode. This procedure is similar to the Hjorth transformation (Hjorth, 1975) but differs in that it averages across neighboring electrode pairs, thereby enhancing the association between surface EEG and underlying perfusion compared with linked-ears or conventional Hjorth referencing.

Second, relative power values were calculated by dividing absolute power by total power at each electrode in the theta frequency band (4–8 Hz). To normalize, the maximum absolute (AMAX*_*f*_*) and maximum relative (RMAX*_*f*_*) power values in theta were identified, and each electrode’s values were scaled accordingly, yielding A*NORM*_(*s,f*)_ and R*NORM*_(*s,f*)_. Third, cordance values were obtained by summing ANORM and RNORM and subtracting a half-maximal reference (0.5):


CORDANCE=(s,f)(A-NORM(s,f)0.5)+(R-NORM⁢(s,f)0.5)

For group-level analyses, electrode theta cordance values were averaged into anatomically defined regions of interest, including prefrontal (Fp1, Fz, Fp2), frontocentral (F3, Fz, F4), central (C3, Cz, C4), left temporal (T3, T5), right temporal (T4, T6), left parietal (P3, P5), right parietal (P4, P6), and occipital (O1, O2) areas. These regional indices served as the primary neurophysiological measures (Leuchter et al., 1994a).

Cordance values were calculated individually for each participant using the original algorithm described by Leuchter et al. (1994a). No additional normalization procedures were applied at the group level; reported group means represent averages of the individual cordance values.

### VAR-based directional predictive analysis

Although Granger causality was originally developed for temporally ordered time-series data and has been widely applied to neuroimaging data (Deshpande et al., 2011; Roebroeck et al., 2005), the present study employs a VAR-based framework as an exploratory method for examining directional predictive dependencies among cross-sectional EEG cordance measures and clinical indices. The resulting estimates should not be interpreted as evidence of temporal causality. Rather, they reflect statistical asymmetries in predictive relationships across the sample. In this framework, “lag = 1” refers to a one-step difference in the ordering of participants after sorting according to a predefined analytical criterion. This does not imply temporal precedence between individuals but instead represents a statistical dependency structure across inter-subject variability. Accordingly, the present analysis is intended to identify directional predictive associations rather than causal effects. Comparable VAR-based approaches have been applied across a range of neural datasets, including cortical electrophysiology and resting-state functional imaging (Guo et al., 2020; Sheikhattar et al., 2018; Wang et al., 2020).

### Statistical analysis

All analyses were performed to characterize the sample and evaluate group differences in demographic, clinical, and neurophysiological measures. Descriptive statistics, including means, standard deviations, and frequency distributions, were computed for demographic and clinical variables. Between-group comparisons of demographic features and regional cordance values were conducted using independent-samples *t*-tests, complemented by Bayesian independent-samples *t*-tests to quantify the strength of evidence for or against group differences. Associations between theta cordance values and clinical variables, including SOGS scores, disorder duration, and psychological symptom measures, were examined using Pearson’s correlation coefficients. All significance tests were conducted at a two-tailed α level of 0.05, and effect sizes were reported using Cohen’s d. Analyses were performed using SPSS (version 27) for descriptive and frequentist statistics and EViews 12 for exploratory vector autoregressive (VAR)-based directional predictive modeling.

## Results

### Demographics and clinical characteristics

A total of 28 patients with GD and 29 HCs were included (all male). The groups differed in raw age distributions (GD: 32.5 ± 10.1 years; HC: 40.2 ± 5.8 years), but analyses were conducted after age-matching to ensure comparability, and no residual age effects were present in the statistical models. Education levels were broadly comparable; the majority of participants in both samples had at least a high school education (89% of GD and 100% of HCs achieved high school or higher). In the GD group, 5 individuals (17.9%) had at least one comorbid psychiatric condition (primarily Attention-Deficit/Hyperactivity Disorder in 3 cases, and one case each of major depression and obsessive-compulsive disorder), whereas no comorbid diagnoses were present in HCs. The GD patients had a mean illness duration of 7.1 ± 4.3 years (range 1–18). Half of the patients (50%) had no children, while the remainder had one or more (up to 3); data on children were not collected for the control group. Overall, the GD and HC groups were well characterized demographically, with the expected clinical differences in gambling-related characteristics (Table 1).

**TABLE 1:** Demographic and clinical characteristics.

Characteristic	GD (*n* = 28)	HC (*n* = 29)
Age, years (Mean ± SD)	32.5 ± 10.1	40.2 ± 5.8
Education ≥ high school, *n* (%)	25 (89.3%)	29 (100%)
Any psychiatric comorbidity, *n* (%)	5 (17.9%)	0 (0%)
Illness duration, years	7.1 ± 4.3 (1–18)	–
Having ≥ 1 children, *n* (%)	14 (50.0%)	–

Data are presented in this table as mean ± standard deviation or *n* (%). Although raw age values differed significantly between groups, analyses were performed after age-matching to minimize confounding. Comorbidity refers to the presence of any co-occurring psychiatric disorder (e.g., ADHD, depression, OCD).

### Theta cordance GD and HC group comparisons

**FIGURE 1:**
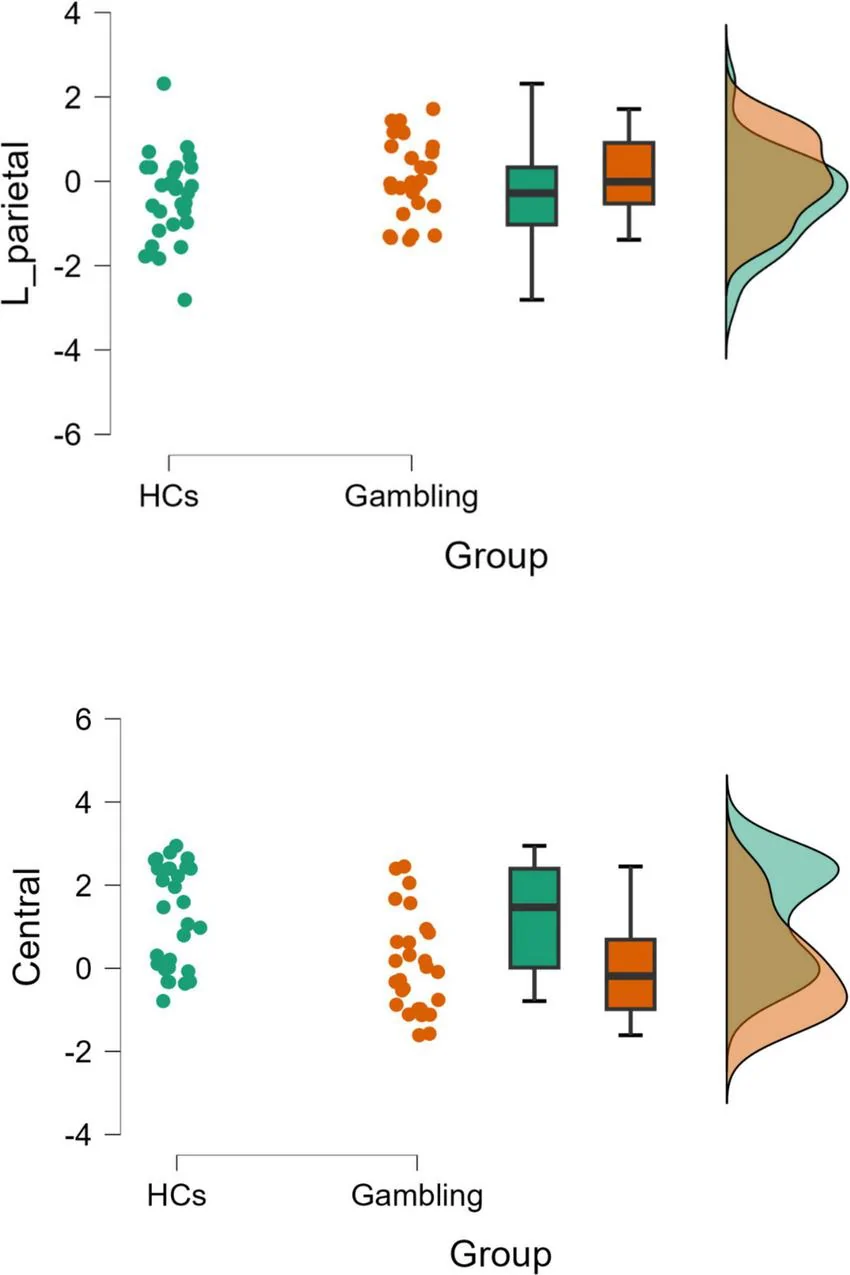
Regional differences in EEG theta cordance between gambling disorder (GD) patients and healthy controls (HCs). Individual observations, boxplots, and density distributions are shown for the two regions demonstrating significant between-group differences (central and left parietal cordance). Boxplots display the median and interquartile range, while density plots illustrate the distribution of values within each group. The plot displays the distribution of individual cordance values, group density estimates, and boxplots. Left parietal cordance was significantly higher in the GD group compared with HCs (GD: 0.13 ± 0.95; HC: –0.43 ± 1.03; *t*(55) = 2.12, *p* = 0.039, Cohen’s *d* = 0.56).

Group-wise comparisons of resting-state theta cordance revealed a significant regional difference between GD patients and HCs. In the central region, GD patients showed markedly lower theta cordance values (Mean ± SD: 0.03 ± 1.18) compared to HCs (1.25 ± 1.23), reflecting a large effect size (Cohen’s *d* = −1.00) and a highly significant group difference (independent *t*-test: t_(55) = −3.79, *p* < 0.001). A significant difference was also observed in the left parietal region: GD patients had higher (less negative) left parietal theta cordance on average (0.13 ± 0.95) than HCs (−0.43 ± 1.03), with a moderate effect size (*d* = 0.56) and statistical significance (t_(55) = 2.12, *p* = 0.039). Trends toward group differences emerged in two additional regions. Occipital theta cordance was marginally elevated in the GD group (0.02 ± 1.71 vs. −0.67 ± 0.75 in HCs), with a trend-level effect (t_(36.7) = 1.96, *p* = 0.052). Similarly, left temporal theta cordance tended to be higher in GD (0.17 ± 0.84) than in HCs (−0.34 ± 1.19), although this difference did not reach significance (t_(50.5) = 1.87, *p* = 0.067). In contrast, prefrontal, frontocentral, right temporal, and right parietal theta cordance values did not differ significantly between groups (all *p* > 0.23, | d| < 0.33; Table 2). Figures 1 illustrate the two regions that showed significant group differences (central and left parietal). Trend-level effects in the occipital and left temporal regions are reported in the text but are not displayed in the figures.

**TABLE 2:** Regional EEG theta cordance in GD patients and healthy controls (HCs), with group comparisons and effect sizes.

Region	GD (Mean ± SD)	HC (Mean ± SD)	t(df)	Cohen’s d	*P*-value
Prefrontal	0.25 ± 1.16	0.21 ± 0.81	0.18 (55)	0.05	0.855
Frontocentral	0.18 ± 0.73	0.33 ± 0.77	−0.74 (55)	−0.20	0.463
Central	0.03 ± 1.18	1.25 ± 1.23	−3.79 (55)***	−1.00	**<0.001**
Left temporal	0.17 ± 0.84	−0.34 ± 1.19	1.87 (50.5)	0.49	0.067
Right temporal	−0.26 ± 1.09	−0.59 ± 0.99	1.20 (55)	0.32	0.234
Left parietal	0.13 ± 0.95	−0.43 ± 1.03	2.12 (55)*	0.56	**0.039**
Right parietal	−0.48 ± 1.08	−0.47 ± 1.18	−0.03 (55)	−0.01	0.973
Occipital	0.02 ± 1.71	−0.67 ± 0.75	1.96 (36.7)	0.53	0.058

In this table, values are mean ± SD. Positive Cohen’s d indicates higher cordance in the GD group. Bold values indicate statistically significant group differences (*p* < 0.05). *p < 0.05, ***p < 0.001.

The observed group differences should be interpreted separately from the directional predictive analyses reported below. Whereas group comparisons characterize average neurophysiological differences between gambling disorder patients and healthy controls, the directional analyses examine associations among variables within the gambling disorder sample itself.

### Correlations between cordance and clinical measures

Within the GD group, Pearson correlation analyses examined the relationships between regional cordance levels and clinical measures, including gambling severity, illness chronicity, and mood symptoms. These analyses did not reveal any significant linear associations. In particular, cordance values in all brain regions were uncorrelated with SOGS scores (a measure of gambling severity); correlation coefficients were low (r ranging from −0.17 to +0.31) and none approached significance (all *p* > 0.10). For example, right temporal theta cordance showed the highest correlation with SOGS (*r* = 0.307), but this was not statistically significant (*p* = 0.112). No meaningful relationships were observed between regional theta cordance and the duration of GD (illness years) or between theta cordance and psychopathology scores (Beck Depression Inventory-II and Beck Anxiety Inventory scores), with all corresponding Pearson r values near zero and non-significant (*p* > 0.20). Thus, in this sample of GD patients, resting EEG cordance measures were not significantly associated with gambling severity, illness chronicity, or concurrent symptoms of depression/anxiety. It is noteworthy, however, that although static correlations were absent, Granger causality analyses subsequently revealed significant directed influences between theta cordance and clinical indices. This apparent discrepancy is expected, since GC assesses predictive and directional dependencies that are not captured by contemporaneous linear correlations, underscoring its added value in identifying brain–behavior dynamics.

### Directional predictive analysis results

#### Gambling disorders group

**FIGURE 2:**
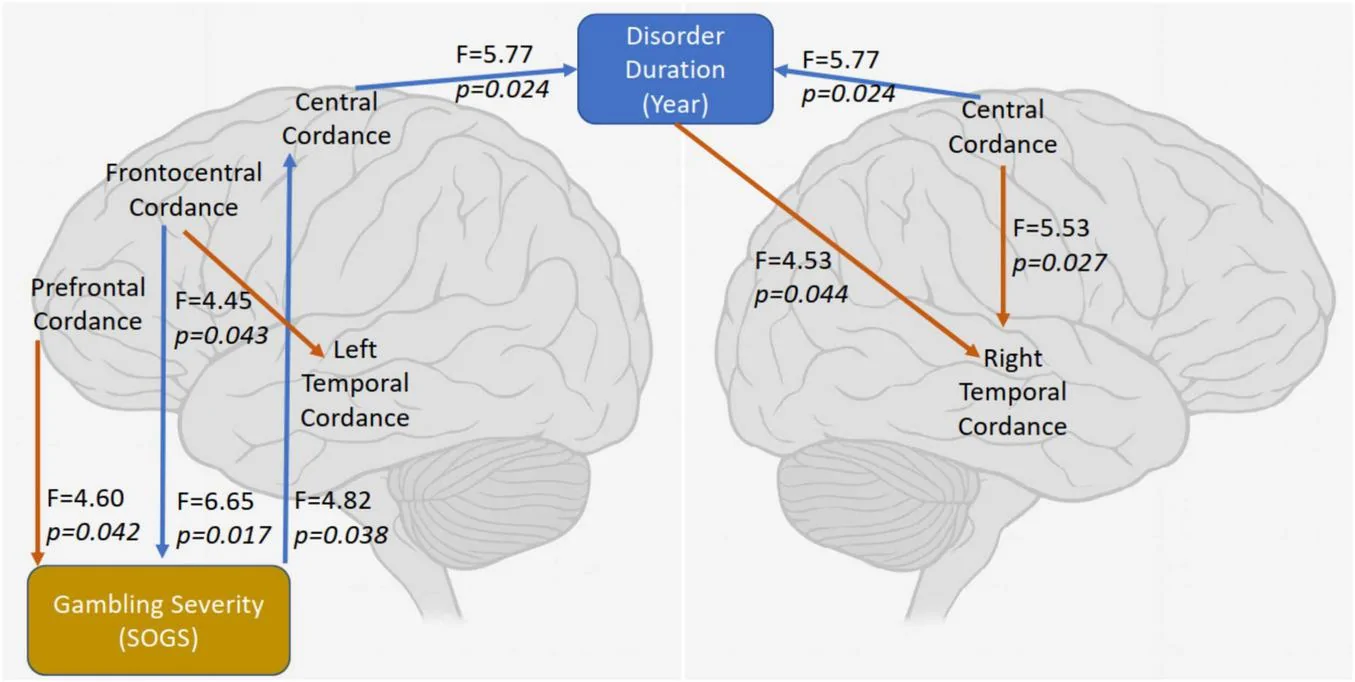
Directional predictive network of EEG theta cordance and clinical indices in gambling disorder (GD).

To explore potential directional relationships between cortical activity and clinical measures in GD, a vector autoregressive model (lag = 1) and pairwise VAR-based directional predictive analysis were applied to the GD group’s data. Despite the cross-sectional design, this analysis identified several significant directed influences between EEG cordance measures and clinical indices (Figure 2). Notably, central theta cordance emerged as a key predictor: central region cordance values showed a significant directional predictive association with right temporal cordance (*F* = 5.53, *p* = 0.027) and also predicted disorder duration (years of gambling disorder; *F* = 5.77, *p* = 0.024). Conversely, illness duration showed a significant directional predictive association with right temporal theta cordance (*F* = 4.53, *p* = 0.044), suggesting a reciprocal predictive relationship within the fitted VAR model.

Model diagnostics were performed to evaluate the adequacy of the VAR specification in the GD sample. Residual autocorrelation testing showed no significant departures from independence (Portmanteau *Q* = 18.6, *p* = 0.23). Examination of the inverse roots of the autoregressive polynomial indicated that all roots lay within the unit circle, supporting model stability. Ramsey RESET tests provided no evidence of substantial non-linear misspecification (all *p* > 0.10). Nevertheless, given the modest sample size (*n* = 28), these findings should be interpreted as exploratory and require replication in larger samples.

In addition, significant directional neural-to-clinical association of frontal regions on clinical severity were found. Prefrontal theta cordance values showed a significant directional predictive association with SOGS scores (gambling severity), with *F* = 4.60, *p* = 0.042. A similar but even stronger effect was observed for the frontocentral theta cordance, which predicted SOGS scores (*F* = 6.65, *p* = 0.017). These findings indicate that higher frontal (especially frontocentral) theta cordance precedes and predicts higher gambling severity, consistent with a potential top-down modulatory role of frontal cortex in driving addictive behavior. SOGS scores also Granger directional predictive association with central theta cordance (*F* = 4.82, *p* = 0.038), completing a reciprocal predictive association wherein greater gambling severity predicts subsequent increases in central region theta cordance. Finally, a significant inter-regional cortical influence was detected: frontocentral theta cordance Granger directional predictive association with left temporal theta cordance (*F* = 4.55, *p* = 0.043), suggesting directed functional connectivity from frontal to temporal regions.

In Figure 2, arrows indicate significant predictive relationships (*p* < 0.05) identified by Granger causality analysis. Frontal hubs (prefrontal, frontocentral) predicted gambling severity (SOGS scores), while central cordance predicted both illness duration and right temporal activity. In turn, gambling severity and illness duration predicted central and right temporal cordance, forming bidirectional loops. Red arrows represent brain-to-clinical influences, blue arrows clinical-to-brain feedback, and black arrows inter-regional cortical links. Node size reflects hub centrality. Directionality reflects statistical prediction within the cross-sectional analytical framework and should not be interpreted as evidence of temporal causality.

#### Healthy control group

For HCs group, VAR lag-order selection criteria were examined using the endogenous variables central, frontocentral, left parietal, left temporal, occipital, prefrontal, right parietal, and right temporal, with a constant term (C) included as an exogenous variable. Based on 29 HC observations, model selection indices [log likelihood, sequential modified LR test, final prediction error (FPE), Akaike information criterion (AIC), Schwarz criterion (SC), and Hannan–Quinn criterion (HQ)] converged on lag = 2 as the most appropriate order. Specifically, LR, FPE, and AIC criteria, as well as HQ, supported the 2-lag specification, whereas only SC suggested lag = 0. Overall, the majority of information criteria indicated lag = 2 as the optimal choice.

**FIGURE 3:**
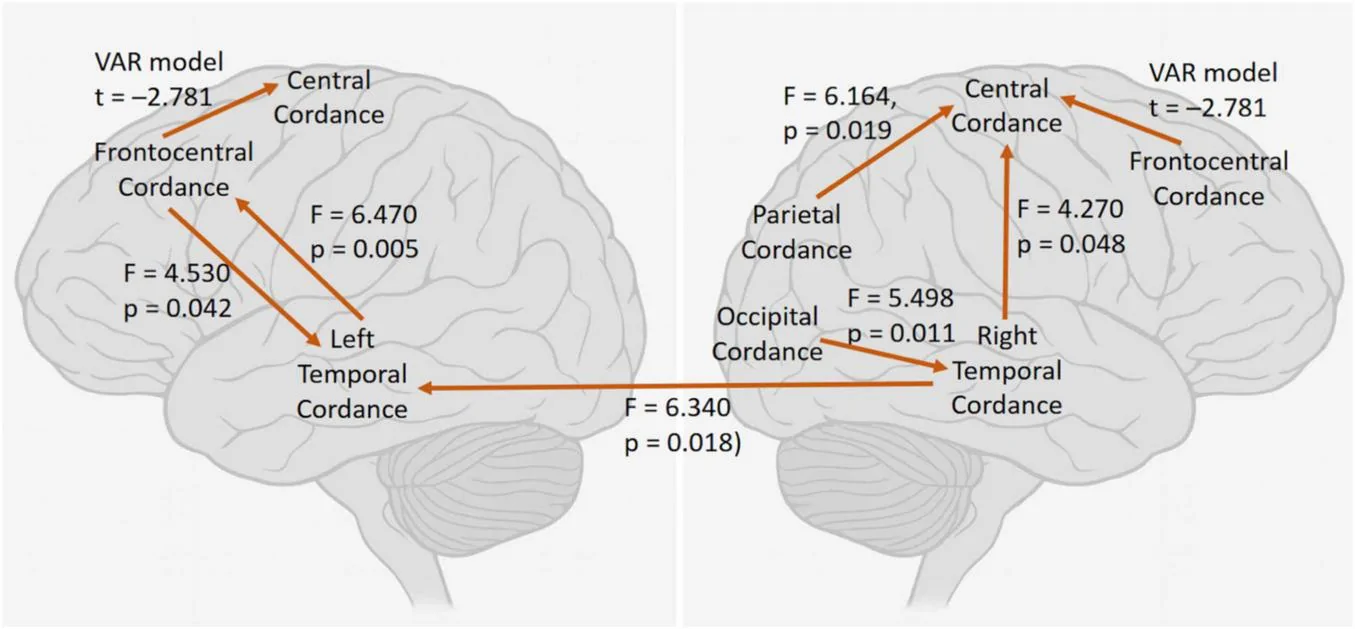
Directed predictive network of EEG cordance in healthy controls.

Granger causality analysis identified four significant directed theta cordance connections in the HC group (Figure 3). These included connections from right parietal to central (*F* = 6.164, *p* = 0.019), from right temporal to central (*F* = 4.270, *p* = 0.048), from frontocentral to left temporal (*F* = 4.530, *p* = 0.042), and from right temporal to left temporal (*F* = 6.340, *p* = 0.018). These findings highlight a prominent role of right-hemisphere parietal and temporal regions in predicting central and left temporal activity. Bidirectional Granger causality tests (lag = 2) further revealed the strongest effect from left temporal to frontocentral (*F* = 6.470, *p* = 0.005), followed by from occipital to right temporal (*F* = 5.498, *p* = 0.011) and from right temporal to left temporal (*F* = 3.646, *p* = 0.041). This indicates that temporal and occipital theta cordance activity significantly predicts frontocentral and contralateral temporal regions, underscoring their role as critical causal hubs in the healthy brain network.

VAR estimations provided additional support for these dynamics. For example, frontocentral theta cordance at lag –1 exerted a significant negative effect on central activity (*t* = −2.781), consistent with frontotemporal integration. Likewise, right temporal theta cordance at lag –1 predicted both central and parietal activity, suggesting that right temporal inputs drive information flow toward central and parietal regions. R^2^ values ranged between 0.56 and 0.81, confirming moderate-to-strong explanatory power, although adjusted R^2^ was lower for certain regions (e.g., left temporal: 0.027), reflecting interindividual variability. Together, these results suggest that in healthy controls, theta cordance networks are characterized by temporo-occipital drivers shaping frontocentral and parietal regions, with right temporal and frontocentral cortices emerging as critical nodes. Unlike the GD group, where central and frontal hubs were tightly coupled with clinical severity, the HC group exhibited a stable physiological organization supporting perceptual and cognitive integration.

As presented in Figure 3, significant causal influences (*p* < 0.05) are shown, with right parietal and right temporal regions emerging as key drivers of central and left temporal activity. Bidirectional connections included from left temporal to frontocentral and from frontocentral to left temporal, along with from occipital to right temporal, highlighting temporo-occipital hubs as critical for information flow. Unlike the GD network, no clinical variables are included; all nodes represent cortical regions. Arrow thickness reflects relative F-statistic strength, with thicker arrows indicating stronger causal influence. Directionality reflects statistical prediction among variables within the cross-sectional framework and does not imply temporal information flow.

## Discussion

Our findings provide evidence for a bidirectional relationship between cortical theta cordance and the clinical severity of GD. Specifically, The VAR-based directional predictive analysis revealed that prefrontal and central cordance values had significant predictive interactions with clinical indices such as South Oaks Gambling Screen scores and illness duration. In one direction, aberrant brain activity in frontal regions predicted gambling severity within the modeled directional framework, supporting a “top-down” effect wherein dysfunctional prefrontal cortex activity may contribute to poorer impulse control and escalating gambling behavior. This is consistent with models of addiction positing that impaired frontal executive control leads to disinhibited, compulsive behavior (Heffernan et al., 2024; Kräplin et al., 2022; Su et al., 2024). Equally important, we observed the reverse influence: clinical state predicted subsequent shifts in EEG cordance, indicating a “bottom-up” effect by which the progression of the disorder (e.g., longer illness chronicity or higher recent gambling involvement) induces measurable neurophysiological changes. It is important to note that these directional influences were detectable with GC despite the absence of significant static correlations between cordance and clinical measures. This apparent discrepancy is methodologically consistent, as correlation reflects only synchronous associations, whereas GC captures predictive dependencies across domains. Such reciprocity suggests a vicious cycle in GD: frontal cortical dysregulation can drive excessive gambling (Yin et al., 2024), and the repeated engagement in gambling in turn feeds back onto brain function (Dong et al., 2021), potentially via maladaptive neuroplastic changes (Dimitropoulou and Baxter, 2024). This interpretation aligns with the idea that addiction is a dynamic process in which brain and behavior continually influence one another, rather than a one-time cause or consequence (Smith, 2021).

An important consideration is the apparent contrast between the group-level and within-group findings. Relative to healthy controls, individuals with gambling disorder exhibited significantly reduced central theta cordance and elevated left parietal cordance, suggesting altered regional cortical perfusion or metabolic activity. At the same time, within the gambling disorder group, higher frontal and frontocentral cordance values showed directional predictive associations with greater gambling severity. These findings are not necessarily contradictory because they reflect different levels of analysis. Group comparisons capture average differences between patients and controls, whereas directional predictive analyses examine variability among patients who already share the disorder. Thus, although gambling disorder as a whole may be characterized by reduced frontal-central cordance relative to healthy individuals, patients with relatively higher frontal cordance within the clinical range may exhibit greater symptom severity. This pattern suggests that distinct neurophysiological mechanisms may underlie disorder presence and disorder severity.

Our *a priori* hypothesis predicted that frontal cortical dysfunction and clinical severity would demonstrate a reciprocal relationship, consistent with models of addiction as a feedback cycle (Eddie et al., 2022). The results confirmed this expectation: frontal (prefrontal and frontocentral) cordance predicted gambling severity, while illness duration and severity in turn predicted subsequent changes in central and temporal cordance. This bidirectional interplay supports the idea that GD is maintained by a dynamic loop of brain–behavior interactions rather than a unidirectional cause–effect pathway (Bussu and Detotto, 2015).

When directional and individual differences are considered, the GD group demonstrated reciprocal interactions linking the frontocentral and central cortical regions with both clinical severity and the right temporal lobe. This pattern suggests that frontal and central regions form a feedback network that both predicts and is shaped by the course of the disorder (Potenza, 2014). In contrast, the HC group exhibited cortical interactions predominantly along from temporal to frontal and from occipital to temporal pathways, with central and frontal cortices supporting stable information integration without direct ties to clinical indices. Whereas GD networks (lag = 1) were characterized by clinically related top-down frontal effects and bidirectional reciprocal predictive association, HC networks (lag = 2) showed no clinical associations, with critical hubs located in temporal and occipital regions. In summary, GD patients displayed top-down (from frontal to clinical) and bottom-up (from illness duration to temporal) interactions closely tied to disorder severity, while in HCs, causality was largely driven by temporo-occipital inputs, supporting perceptual and cognitive processes. These findings highlight that GD patients develop a pathological modulation network centered on frontal and central hubs (Piccoli et al., 2020), which contrasts with the normative, perceptual–cognitive organization in healthy brains (Potenza et al., 2019).

The Granger causality results from HCs further underscore that functional network organization is primarily rooted in temporal, occipital, and frontocentral interactions. A strong left temporal → frontocentral influence is consistent with prior reports linking temporal sources to frontal executive areas during language, memory, and auditory processing tasks (Friederici, 2011; Vigneau et al., 2006). Likewise, from occipital to right temporal causality aligns with visual information driving temporal-lobe object and face recognition processes (Beauchamp, 2005; Haxby et al., 2000). The observed from right temporal to left temporal influence points to interhemispheric coordination, which is critical for language, music perception, and memory (Giraud and Poeppel, 2012). Together, these results suggest that in healthy controls, temporo-frontal and occipito-temporal interactions represent fundamental causal drivers, supporting the neural dynamics underlying perception, cognition, and language.

These results extend prior research on the neural correlates of GD by introducing a directional dimension that has been largely absent. Previous studies have shown frontal lobe dysfunction and abnormal connectivity in gamblers but have relied on cross-sectional or undirected measures (e.g., coherence, correlations, spectral power), which cannot establish causal precedence (Bellmunt-Gil et al., 2023; Cavedini et al., 2002; Ding et al., 2013; Jin et al., 2016). Our application of a directional predictive modeling framework directly addresses this gap by testing predictive influences between brain and behavior. The finding of bidirectional links is methodologically innovative, moving beyond correlations to identify reciprocal predictive association. To the best of our knowledge, this is the first application of a VAR-based directional predictive framework modeling to theta cordance in a behavioral addiction, opening a new avenue for studying effective connectivity in addiction psychophysiology.

The demonstration of top-down and bottom-up influences in GD aligns with models of maladaptive neuroplasticity in addiction (Kalivas and O’Brien, 2008; Madsen et al., 2012; O’Brien, 2009). Repeated gambling may strengthen rewards-related pathways while weakening prefrontal inhibitory control (Mestre-Bach and Potenza, 2024). Our findings empirically support this mechanism, showing that greater gambling severity is linked with cortical deviations that impair executive control. The involvement of prefrontal regions is particularly noteworthy, as abnormal frontal activity predicted worsening clinical scores (Balodis et al., 2012). Conversely, clinical severity also predicted subsequent cordance changes, suggesting that ongoing behavioral engagement remodels brain function. Neuroimaging studies have similarly shown heightened limbic responses to gambling cues (e.g., amygdala, insula), which may further sensitize rewards circuits and diminish frontal regulation (García-Castro et al., 2023).

Beyond their theoretical significance, these results carry promising translational implications. If EEG theta cordance measures and clinical severity are truly entwined in a reciprocal predictive association, then cordance might serve as an accessible neurobiomarker for monitoring disorder progression or recovery. Theta cordance is obtained non-invasively and reflects underlying cerebral perfusion/metabolism; thus, longitudinal tracking of theta cordance could potentially alert clinicians to impending changes in a patient’s clinical state. For example, a drop in prefrontal theta cordance (indicating reduced frontal activity) might Granger-cause a future spike in gambling urges or relapse severity, suggesting that such an EEG change could be an early warning sign of relapse risk. Conversely, improvements in clinical status (through abstinence or therapy) might eventually drive normalization of theta cordance values, which could be used to objectively gauge treatment response. This kind of brain–behavior dynamical analysis could pave the way for personalized interventions. In patients where brain-to-behavior predictive relationship is dominant, treatments enhancing frontal cortical function – such as neuromodulation or cognitive training to improve executive control – might be especially effective. In cases where behavior-to-brain effects prevail, strategies targeting behavioral change (e.g., environmental modifications, cue-exposure therapies or medications reducing rewards sensitivity) could help interrupt the cycle and allow the brain to recover normal function. Importantly, cordance-based Granger causality analyses might help stratify patients or predict outcomes, analogous to how prefrontal theta cordance has been investigated as a biomarker for antidepressant treatment response in major depression. By applying similar logic to GD, we can envision using cordance and its directional relationships with symptoms to identify who is likely to benefit from certain interventions or to develop real-time monitoring tools. Ultimately, the integration of theta cordance with directional causality analysis represents a step toward translational neurophysiology in GD – leveraging quantitative brain metrics to inform clinical decision-making. Future studies should expand on this approach, potentially combining theta cordance with other modalities (such as fMRI or neurochemical imaging) to validate its sensitivity, and exploring whether modifying the detected brain–behavior loops (for instance, via neuromodulatory treatments) can effectively reduce gambling pathology.

### Limitations

This study has several limitations. First, the sample size was modest and restricted to male patients, which may limit statistical power and the generalizability of the findings to broader populations. Although several directional predictive associations reached statistical significance, smaller effects may have gone undetected. Second, the retrospective and cross-sectional design precludes conclusions regarding temporal or causal relationships between cortical activity and clinical severity.

Third, the present directional predictive analysis was applied to cross-sectional data and therefore treats participants as ordered observations rather than true temporal measurements. While model diagnostics supported the adequacy and stability of the fitted VAR models, this approach remains non-standard and assumes that inter-subject variability captures meaningful variation in the underlying brain–behavior system. Consequently, the identified directional associations should be interpreted as predictive statistical dependencies rather than evidence of temporal causality.

Fourth, the interpretation of lag structure in the present analyses is not temporal. Although cross-sectional VAR-based approaches may identify directional predictive relationships among variables, they cannot establish within-subject temporal dynamics. Longitudinal studies with repeated EEG assessments are required to determine whether the observed associations reflect genuine temporal brain–behavior processes.

Finally, all data were obtained from a single clinical site, and replication in larger, more diverse, and longitudinal cohorts will be necessary to establish the robustness and generalizability of the present findings.

### Clinical implications

The identification of bidirectional brain–behavior interactions in GD suggests that EEG cordance may serve as a non-invasive biomarker for monitoring disease progression and treatment response. Frontal theta cordance changes predicting gambling severity point to the potential utility of cordance in relapse risk assessment. Conversely, the influence of clinical severity on subsequent cordance highlights the possibility of tracking neural adaptations during behavioral or pharmacological interventions. These findings support the development of personalized treatment strategies, where neurophysiological monitoring could help guide intervention timing and modality. Ultimately, integrating cordance-based directional predictive modeling into clinical settings may facilitate real-time monitoring and personalized treatment in GD and related addictions.

## Conclusion

This study provides the first evidence that cortical cordance and clinical severity in GD are dynamically and reciprocally linked. VAR-based directional predictive modeling identified both directional neural-to-clinical association of frontal cortical dysfunction on gambling severity and directional clinical-to-neural association of clinical state on subsequent neural activity, suggesting a self-reinforcing cycle of brain–behavior interactions. These findings extend current models of addiction as maladaptive neuroplasticity and highlight directional predictive modeling based on EEG theta cordance as a promising tool for developing biomarkers and informing personalized interventions in behavioral addictions.

## Data Availability

The raw data supporting the conclusions of this article will be made available by the authors, without undue reservation.
